# Gamma Knife-Mimicking Radiosurgery With Cyberknife: A Comparison of Dose Prescription Methodologies for the Treatment of Trigeminal Neuralgia

**DOI:** 10.7759/cureus.99223

**Published:** 2025-12-14

**Authors:** Olivier Blasi, Krishna Monroe, Chase Mallory, Ronald Hammers, Alan Monroe

**Affiliations:** 1 Medical Physics, Colorado Associates in Medical Physics, Colorado Springs, USA; 2 Medical Oncology, Georgia Institute of Technology, Atlanta, USA; 3 Neurosurgery, AdventHealth Parker, Parker, USA; 4 Radiation Oncology, Penrose Cancer Center, Colorado Springs, USA

**Keywords:** cyberknife, gamma knife, meckel's cave, stereotactic radiosurgery, trigeminal neuralgia

## Abstract

Objective: Cyberknife radiosurgery can be prescribed to a longer, volumetric segment of the trigeminal nerve or using an isocentric technique that mimics the spherical distribution of the Gamma Knife. This study aimed to compare these two distinct radiosurgical planning approaches.

Methods: The authors performed a retrospective review of consecutive patients treated between 2010 and 2022 with Cyberknife radiosurgery for trigeminal neuralgia (TN). Dosimetric analysis was performed to investigate the relationship between pain response and various dose parameters to the trigeminal nerve, brainstem, and Meckel’s cave.

Results: One hundred forty-two patients were studied: 100 with type I TN and 42 with type II. The initial 55 patients (cohort 1) received a median dose of 60 Gy and a maximum dose of 74.7 Gy prescribed to a roughly 6 mm volume contoured along the cisternal segment of the trigeminal nerve. The subsequent 87 patients (cohort 2) were treated to a maximum dose of 85 Gy prescribed with an isocentric approach, resulting in a spherical dose distribution. Initial adequate pain relief was seen in 88% of patients overall. Type I patients had more favorable outcomes both initially (94% vs. 74%; p=0.0015) and at last follow-up (62% vs 36%; p=0.0055) compared with type II patients. Complete pain response (BNI I) was seen in 42% of cohort 1 patients and in 33% of cohort 2 patients (p=0.3574).

Conclusions: Volumetric stereotactic radiosurgery (SRS) prescription to a lower dose/longer segment of nerve and isocentric prescription to a higher dose/tighter volume are both acceptable options for treatment of TN.

## Introduction

Trigeminal neuralgia is an often-debilitating neurologic pain syndrome frequently characterized by paroxysms of pain in the distribution of the trigeminal nerve. Treatment options include medical management for most patients, with microvascular decompression (MVD), percutaneous procedures, and stereotactic radiosurgery (SRS) being reasonable options for those who are refractory to pharmacologic manipulation [1]. Numerous retrospective publications over the past few decades have been consistent in showing the effectiveness of SRS in the treatment of medically refractory trigeminal neuralgia [2-13]. Much of the recent practice-defining literature is centered around the Gamma Knife unit [2,4,5,7-9,12-14], with Cyberknife [3,6,10-11] and linear accelerator (LINAC)-based radiosurgery [15,16] reports showing similar encouraging results, albeit with smaller patient numbers and shorter follow-up in most instances.

While this article was previously presented as a meeting abstract at the 2025 ASTRO Annual Scientific Meeting on September 27, 2025, comparative analyses of specific radiosurgical techniques remain relatively sparse. Target placement is variable in the literature, with many series targeting the dorsal root entry zone (DREZ), while others have shown excellent results targeting closer to the retrogasserian zone (RGZ) [17]. Target shapes range from spherical to cylindrical, depending mostly on the type of unit delivering the treatment.

When our radiosurgical program was being established in 2009, it was modelled after the primary Cyberknife experience published at the time from Stanford. The technique described by Adler et al. involved “spilling dose into the ambient cistern” along the nerve and maintaining relative homogeneity within target volumes that were roughly cylindrical in shape [18]. Their reported marginal prescription doses were 56-62 Gy, prescribed on average to the 79% isodose line, with maximum doses of approximately 75 Gy.

As excellent results continued to be published from Gamma Knife centers, treating generally higher maximum doses to smaller spherical volumes, we opted to alter our dose prescription and treatment planning approach in early 2016 toward plans that intentionally mimicked this type of distribution while still using the Cyberknife unit. The present study does not intend to compare radiosurgery delivery devices. Rather, the reason for this study is to take advantage of our unique experience treating trigeminal neuralgia with two distinct radiosurgical planning approaches to determine the potential benefits of one methodology versus the other.

## Materials and methods

A retrospective review of all cases treated with Cyberknife radiosurgery for trigeminal neuralgia between 2010 and 2022 was performed after obtaining IRB approval. Patients were excluded if they had received prior radiosurgery at an outside facility (seven) or had tumor compression on the trigeminal nerve (two) as a competing diagnosis. The remaining 142 patients comprise the study population.

Institutional treatment planning details have been previously reported [19]. Briefly, immobilization involved a custom-fabricated thermoplastic mask. CT simulation was performed in the treatment position with a 1 mm slice thickness. A stealth-sequence, heavily T2-weighted MRI with 0.8 mm cuts was then obtained for treatment planning, and images were fused in the MIM workstation to permit target delineation and normal structure contouring. Treatment planning occurred within the Accuray MultiPlan workstation for delivery on the Cyberknife G4 robotic SRS system.

Dose prescriptions and targeting methodologies evolved over the study period. The early experience (cohort 1), modeled on the early Stanford experience, involved treating a roughly 6 mm length of the retrogasserian segment of the trigeminal nerve, truncated at least 2 mm from the dorsal root entry zone at the brainstem. Dose was prescribed (57-64 Gy) to cover an oval-shaped volume contoured along the track of the trigeminal nerve, typically to the 70-80% isodose line. This resulted in maximum doses in the range of 70-80 Gy.

**Figure 1:**
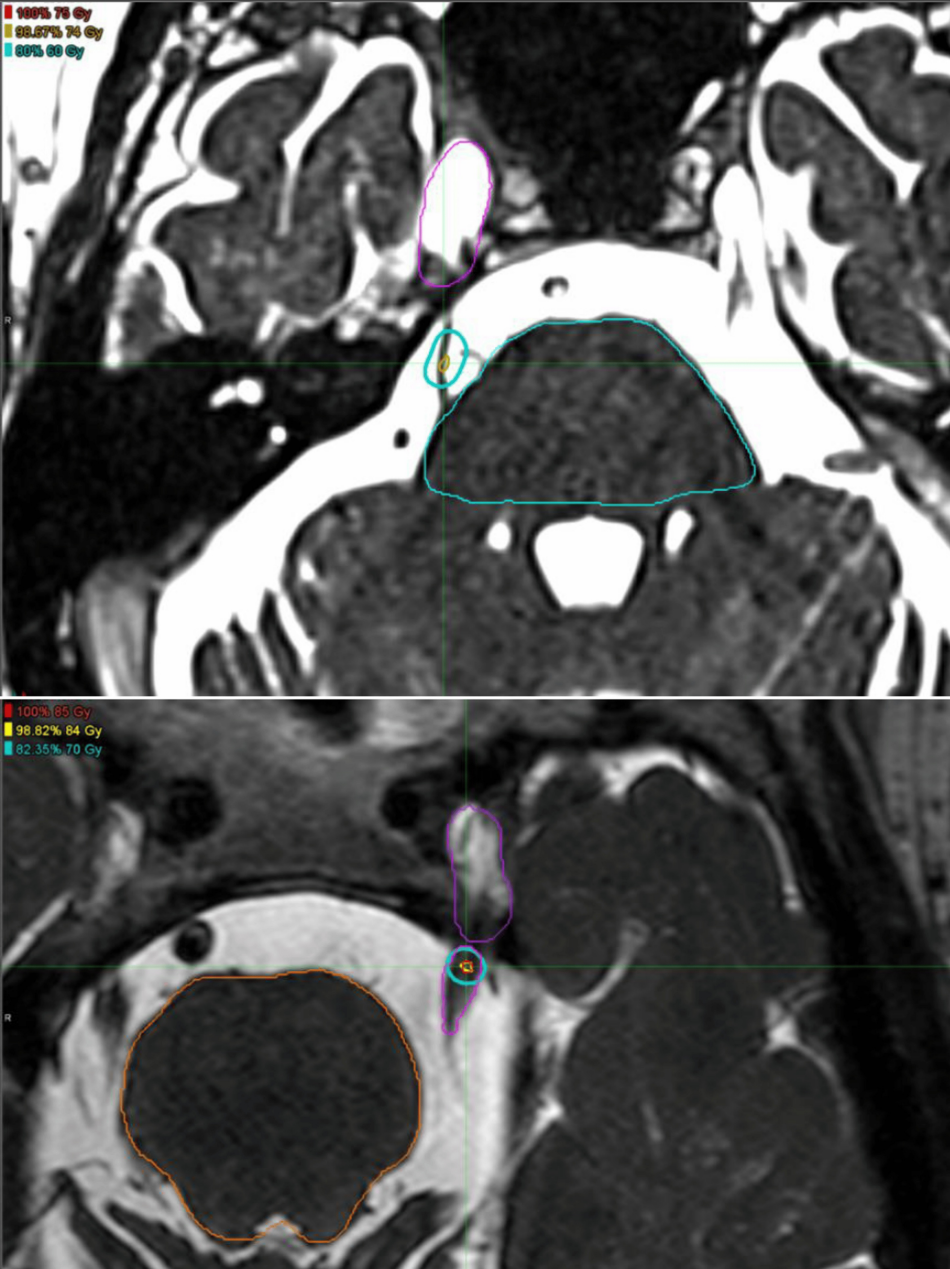
Representative treatment plans from cohort 1 compared with those from cohort 2 The upper image is an example of a typical cohort 1 plan with an oval-shaped 60 Gy isodose line and a maximum dose of 75 Gy. The lower image is an example of a typical cohort 2 plan; the 70 Gy isodose line is spherical in shape, and the maximum dose is 85 Gy.

In early 2016, target delineation was changed from covering a specified volume to an isocentric approach targeting the trigeminal nerve either 2-3 mm anterior to the dorsal root entry zone or in a retrogasserian location, depending on assessment of nerve and brainstem anatomy. Dose was subsequently prescribed to the maximum point dose at the centroid of a contoured spherical volume, resulting in a dose distribution that mimicked published Gamma Knife experiences (cohort 2). This allowed maximum dose escalation up to 85 Gy in nearly all patients in the second cohort; seven patients were prescribed 80 Gy around the time of the transition. Figure 1 demonstrates representative treatment plans from cohort 1 compared with those from cohort 2.

Brainstem and Meckel’s cave were both contoured prospectively in each case. Brainstem maximum dose was limited to 50% of the prescription dose. As specific goals for dose to Meckel’s cave did not exist, plans were optimized to keep dose as low as reasonably possible for this structure, provided that brainstem constraints and target coverage goals were met. The cisternal segment of the ipsilateral trigeminal nerve was subsequently contoured between the brainstem and Meckel’s cave for the present analysis.

Clinical variables were recorded from review of medical records and treatment planning documents. Gender, age, presence of prior procedures, and type I vs type II trigeminal neuralgia were recorded as possible explanatory clinical variables. Additional treatment-related variables recorded included prescription dose, maximum dose, various Meckel’s cave and brainstem dosimetry parameters (maximum, 0.03cc, 0.1 cc, and mean), trigeminal nerve length, treatment cohort, and development of treatment-related numbness. The minimum distances between the centroid of the contoured prescription volume and the brainstem and Meckel’s cave were calculated in the MIM workstation. These distances served as objective measures of target location with respect to the critical structures for subsequent analysis. To investigate how the size and shape of the isodose distribution affected outcomes, contoured ipsilateral trigeminal nerve dosimetry was collected, including trigeminal nerve mean dose and percent of the trigeminal nerve receiving 40, 50, 60, 70, and 80 Gy.

The Barrow’s Neurological Institute (BNI) scale was utilized to objectify responses as per recent literature [20]. All patients had BNI scores of IV or V at presentation. The best initial response post SRS was recorded based upon review of clinic records. If patients subsequently had recurrence of pain, the date and BNI level of pain at recurrence were recorded. Finally, the BNI level at the last available follow-up was recorded. Initial adequate pain relief was defined per the existing literature as achieving a BNI score of I-IIIb post treatment [6]. ‘Favorable’ pain response at last follow-up equated to BNI levels I-IIIa [20]. For the small cohort of patients treated bilaterally (10 patients), only the ipsilateral side was considered for analysis. Patients were not excluded from assessment of long-term pain control if they were retreated.

Patients were routinely called by the Cyberknife nurse coordinator within the first week of treatment to assess for toxicity and early pain response assessment. Follow-up then alternated between neurosurgery and radiation oncology; the first visit with neurosurgery was at one month, and the next visit with radiation oncology was at two months post procedure. Beyond the early assessment period, patients typically alternated visits every six months to include both treatment specialists, as well as with neurology, who managed tapering of pain medications (with specific instructions to taper no sooner than at a successful two-month follow-up visit).

Statistical analysis was carried out by an independent biostatistician. Univariate analysis of clinical and dosimetric variables was run for a variety of pain control outcomes. Logistic regression was run for continuous prognostic variables and Fisher’s exact test for discrete variables. The Wilcoxon/Kruskal-Wallis test with one-way chi-square was utilized to compare time to events.

## Results

Table 1 compares clinical demographics and dosimetric parameters between the cohorts. The median follow-up for patients in cohort 1 and cohort 2 were 5.3 years (range: 0.1-13.5 years) and 2.4 years (range: 0.2-7.5 years), respectively.

**Table 1:** Patient demographics

Variables	Cohort 1	Cohort 2
Number	55	87
Average Age	71	66
Gender (M:F)	16:39	30:57
Type I	43	57
Type II	12	30
Prior procedure	10/55	15/87
Multiple sclerosis	5	5
Median Rx dose	60 Gy	85 Gy
Max dose	74.7 Gy	85 Gy
Rx Isodose line	81%	100%
Average Beams	95	101
Average Nodes	82	72

Initial response was seen in 88% of patients overall. Initial pain relief was similar between cohort 1 and cohort 2 (89% vs. 87%; p=0.9999). Complete pain response (BNI I) was seen more frequently in cohort 1 (42%) than in cohort 2 (33%), but the difference was not statistically significant (p=0.3574). Favorable initial pain responses (BNI I-IIIa) were seen in 69% of cohort 1 patients and in 59% of cohort 2 patients (p=0.2309). Table 2 shows initial pain responses for each cohort and by type I vs. type II. 

**Table 2:** Initial response BNI: Barrow’s Neurological Institute

BNI response	Cohort 1 (N=55)	Cohort 2 (N=87)	Type I (N=100)	Type II (N=42)	Total (N=142)
I	23 (42%)	29 (33%)	46 (46%)	6 (14%)	52 (37%)
II	-	2 (2%)	1 (1%)	1 (2%)	2 (1%)
IIIa	15 (27%)	20 (23%)	28 (28%)	7 (17%)	35 (25%)
IIIb	11 (20%)	25 (29%)	19 (19%)	17 (40%)	36 (25%)
None	6 (11%)	11 (13%)	6 (6%)	11 (26%)	17 (12%)

Type I patients (oval-shaped dose distribution) had more favorable outcomes both initially and at last follow-up compared with type II patients (spherical-shaped dose distribution). Initial response was seen in 94/100 (94%) of type I cases and in 31/42 (74%) of type II cases (p=0.0015). Similar difference was maintained (62% vs. 36%; p=0.0055) at the last follow-up. Table 3 demonstrates response rates between the cohorts separated by type I vs. type II. There were too few multiple sclerosis patients (five in each cohort) for statistical comparison, but pain responses approximated those for the type II cohort (BNI 1-IIIa 40%).

**Table 3:** Response by type and cohort Initial and follow-up percentage of patients with corresponding BNI (Barrow Neurological Institute) pain intensity score reported.

Time Point and BNI Score	Type 1 Cohort 1 (N=43)	Type 1 Cohort 2 (N=57)	Type II Cohort 1 (N=12)	Type II Cohort 2 (N=30)
Initial Response I	20 (47%)	26 (46%)	3 (25%)	3 (10%)
Initial Response II	0 (%)	1 (2%)	0 (%)	1 (3%)
Initial Response IIIa	14 (33%)	14 (25%)	1 (8%)	6 (20%)
Initial Response IIIb	6 (14%)	13 (23%)	5 (42%)	12 (40%)
Initial Response None	3 (7%)	3 (5%)	3 (25%)	8 (27%)
Last Follow-up I	12 (28%)	18 (32%)	3 (25%)	3 (10%)
Last Follow-up II	0 (%)	1 (2%)	0 (%)	3 (10%)
Last Follow-up IIIa	11 (26%)	20 (35%)	1 (8%)	5 (17%)
Last Follow-up IIIb	12 (28%)	12 (21%)	3 (25%)	11 (37%)
Last Follow-up IV-V	8 (19%)	6 (11%)	5 (42%)	8 (27%)

In those who developed recurrence after initially successful radiosurgery, the median time to recurrence was 17.3 months in cohort 1 and 8.7 months in cohort 2 (p=0.0645). Repeat radiosurgery was pursued in 11 cohort 1 patients and in three cohort 2 patients. Salvage (non-radiosurgery) procedures were performed in eight cohort 1 patients and in seven cohort 2 patients.

Favorable pain response (BNI I-IIIa) at last follow-up revealed somewhat better numerical rates of ultimate control in cohort 2 (58%), compared with cohort 1 (49%), but the difference was not significant (p=0.3883). Overall pain response (BNI I-IIIb) was 81% overall. Table 4 shows pain responses at the last follow-up for each cohort and by each type. The maximum dose dichotomized by less than or equal to 85 Gy did not affect the overall population of patients, but for type I patients, higher doses resulted in more favorable BNI outcomes at the last follow-up (p=0.0252).

**Table 4:** Pain response at the last follow-up BNI: Barrow’s Neurological Institute

BNI score at the last follow-up	Cohort 1 (N=55)	Cohort 2 (N=87)	Type I (N=100)	Type II (N=42)	Total (N=142)
I	15 (27%)	21 (24%)	30 (30%)	6 (14%)	36 (25%)
II	0 (0%)	4 (5%)	1 (1%)	3 (7%)	4 (3%)
IIIa	12 (22%)	25 (29%)	31 (31%)	6 (14%)	37 (26%)
IIIb	15 (27%)	23 (26%)	24 (24%)	14 (33%)	38 (27%)
IV-V	13 (24%)	14 (16%)	14 (14%)	13 (31%)	27 (19%)

Treatment-related numbness occurred in 20/54 (37%) of cases in cohort 1 at a median time of 16 months. Numbness affected 39/83 at-risk patients (47%) in cohort 2 at a median time of 10.4 months (p=0.1864). Numbness was BNI grade II in most cases (52/59, 88%); grade III numbness was reported in only seven patients, and higher-grade toxicity/anesthesia dolorosa was not seen. Other toxicities included a single case each of corneal abrasion, dry eye syndrome requiring drops, neurotropic keratopathy, hemi-facial spasm, and hyperhidrosis/Frey’s-like syndrome. Development of numbness was associated with complete pain response to treatment (0.0415), and favorable BNI scores at the last follow-up (p=0.0012).

Target location, as measured by the distance between the centroid of the treatment volume and the brainstem/Meckel’s cave, was noted to have an impact on long-term pain control. Larger centroid to Meckel’s cave distance was associated with better pain control at the last follow-up (p=0.0287). For the subset of type I patients, a larger centroid to Meckel’s cave distance was also associated with improved complete pain response (p=0.0418).

Comparing trigeminal nerve dosimetry parameters between the two cohorts, only V40 was significantly different (p=0.0329). All studied volumetric dose coverage parameters for the trigeminal nerve failed to reveal significant differences with respect to pain outcome measures.

Table 5 reports the statistically significant variables for complete pain response and favorable pain response at the last follow-up.

**Table 5:** Univariate analysis of factors associated with pain control (only significant p-values)* * All other variables tested failed to correlate with the outcomes in a statistically significant manner

Variable	Complete response BNI=I (all pts)	BNI I-IIIa at last follow-up (all pts)	Complete response BNI=I (Type I)	BNI I-IIIa at last follow-up (Type I)
Gender	p= 0.0329	*	p=0.0076	*
Max dose (= 85 Gy)	*	*	*	p=0.0252
Prior procedure	p=0.0368	p=0.0160	*	*
Type I vs II	p=0.0057	p=0.0055	N/a	N/a
Numbness	p=0.0415	p=0.0030	*	p=0.0060
Age	p=0.0007	*	p=0.0060	p=0.0231
Distance to Meckel’s	*	p=0.0287	p=0.0418	*

## Discussion

Stereotactic radiosurgery is a well-established, effective tool for the treatment of medically refractory trigeminal neuralgia, irrespective of the type of machine used to deliver the dose. Nevertheless, there are differences in technique that have not previously been directly compared, owing to the retrospective nature of much of the trigeminal neuralgia literature and the generally prohibitive cost and redundancy of owning multiple radiosurgical delivery devices.

The present study demonstrates that both treatment planning paradigms can lead to acceptable outcomes. Those in cohort 1 experienced a somewhat longer time to recurrence when treatment failed and a higher initial favorable response rate relative to cohort 2, but neither finding was statistically significant. These effects could be related to prescribing moderate doses to a longer segment of the trigeminal nerve, though specific volumetric coverage targets for the trigeminal nerve were not appreciated. Cohort 2 patients had somewhat higher pain control rates at last follow-up, and in the subset of type I patients, a maximum dose of 85 Gy was significantly correlated with better long term pain control. These findings seem to confirm a role for higher maximum dose with respect to treatment durability, as has been previously reported. Generally, both techniques were associated with very good outcomes, and it is not possible to declare one technique definitively better than the other.

Table 6 reviews the most notable publications from the past 15 years that specifically reported pain response data and rates of post-radiosurgery numbness [2-11,13-16,20-24]. Pain efficacy is reported using a variety of methods, ranging from subjective response rates to grouped BNI scale measures. Initial pain responses are seen in approximately 80-90%, and response rates fall off by about 20-30% over the next few years. Long term data are generally lacking in most series; however, the Marseille group reported a 45% 10-year rate of pain control, and the Pittsburgh group reported 30% pain control at 10 years.

**Table 6:** Review of the literature

Institution	Date	Device	#Pts	Max Dose (Gy)	Early Efficacy	Late efficacy	Numb
Pittsburgh ^2^	2010	GK	503	80	89%	30%	11%
Netherlands ^21^	2010	GK	450	80	75%	58%	35%
Toronto ^22^	2012	GK	145	80	85%	66%	30%
Wake Forest^ 13^	2012	GK	777	88	86%	60%	27%
Spokane ^23^	2013	GK	108	86	71%	n/a	19%
Georgetown ^5^	2014	GK	36	90	81%	67%	28%
Cleveland Clinic ^4^	2016	GK	870	86	86%	81%	41%
Madrid ^8^	2016	GK	117	90	94%	81%	32%
NYU ^24^	2016	GK	58	80	89%	51%	24%
Marseille ^9^	2016	GK	497	85	92%	45%	21%
Montreal (CHUM) ^3^	2022	CK	166	80	87%	50%	26%
Taipei^ 14^	2018	GK	108	90	90%	70%	55%
Loma Linda ^7^	2019	GK	63	80-85	n/a	68%	21%
Tokyo ^20^	2023	GK	249	90	83%	58%	24%
Stanford ^11^	2009	CK	46	73.5	97%	74%	39%
Milan ^10^	2019	CK	496	71	92%	76%	20%
Italy/Germany ^6^	2020	CK	262	72	91%	71%	18%
UCLA ^15^	2021	LINAC	234	90	93%	72%	32%
Rush ^16^	2017	LINAC	18	90	78%	n/a	44%
Present series	2024	CK	142	85	88%	81%	43%

Among the earliest published Cyberknife reports is one from Romanelli et al. in 2003 [25]. They initially treated an 11 mm nerve segment to 65-70 Gy and noted high rates of bothersome numbness. Subsequently, the same author reported the largest Cyberknife series in the literature, consisting of 527 patients treated to a 6 mm elongated retrogasserian target 10. Results with this approach were excellent, with 92% achieving pain relief at six months and 76% experiencing stable benefit three years after treatment.

Controversy remains regarding the extent of nerve length that needs to be treated for optimal outcomes. Wolf et al. investigated the dose-volume relationship of the trigeminal nerve, reporting that a smaller volume of nerve (<0.35% of nerve volume) receiving a higher dose was associated with fewer pain recurrences [24]. They report a median ratio of 0.55 for the distance from brainstem to isocenter compared with the nerve length, but this surrogate for target location was not predictive of pain response.

Zhao et al. from Huazhong, China, recently described a two-isocenter approach for Gamma Knife SRS [26]. A 4 mm isocenter was placed adjacent to the dorsal root entry zone, and another was placed in the distal cisternal segment. This resulted in a dumbbell-shaped distribution that approximated the early Cyberknife plans in extent of coverage but maintained maximum doses in the range of 84-90 Gy. Initial pain relief was reported in 88%, and an impressive 64% achieved a BNI score of I. Response was durable with an average pain-free time of 49.7 months. These results run counter to those of an early randomized study by Flickinger et al., showing that longer nerve length treated with two isocenters did not significantly improve pain relief but did increase complications [27]. Kienzler et al. compared 4 mm vs. 5 mm collimators in a series of LINAC-based SRS patients. They report better pain control with the larger frame-based 5 mm cohort relative to the 4 mm mask-based cohort [15].

Similar uncertainty surrounds target location placement. Initial work by Matsuda et al. reported better rates of initial complete pain remission targeting the DREZ rather than the gasserian ganglion [28]. Park et al. compared RGZ targeting with DREZ targeting using Gamma Knife treatment planning [17]. Like the current study, their investigation involved a comparison of sequential cohorts treated in distinct methods; early patients were treated with DREZ, and subsequent patients were treated with RGZ. Complete pain relief was more common with RGZ targeting, and the time to response was shorter. Bothersome facial numbness and dry eye symptoms were numerically lower in the RGZ cohort. A study from Milan treating RGZ locations reported better pain relief when the shot-pons distance was < 8 mm [29]. Hung et al. from Taipei failed to see pain response differences based on shot to brainstem distance or nerve length to targeting length ratio [30].

The current study suggests a potential benefit to target placement closer to the brainstem than to Meckel’s cave. Larger centroid to Meckel’s distances correlated favorably with complete response rates in type I patients and in late pain control for the population as a whole. Optimal target location will depend in part on patient anatomy and in part on planning parameters, such as total dose and expectations for coverage of the trigeminal nerve. The present study suggests a benefit to placing the target farther from Meckel’s cave, but it cannot make claims about the relative benefit of DREZ location vs. a mid-nerve location.

Retrospective data best serve in the generation of hypotheses. One could reasonably hypothesize that treatment volume and maximum dose may interact to achieve an optimal plan, and that their effects evolve at different time frames during the follow-up period. With maximum dose showing both consistent importance across the SRS literature and benefit in this dataset, we continue to recommend a maximum dose of at least 85 Gy. The moderate dose isodose distribution could be at play for planning optimization. Based on potentially longer time to recurrence and favorable early responses for cohort 1 in this study, it may be reasonable to allow somewhat greater coverage of the trigeminal nerve if brainstem and other normal structures can be adequately protected. Such a distribution could be achievable with a two-isocenter Gamma Knife plan or by forcing more robust moderate dose coverage with Cyberknife planning. An additional study will be necessary to confirm that such planning leads to better outcomes.

There are inherent weaknesses in any retrospective review. Heterogeneity of dose and target location makes comparison with other series difficult, but this same feature also permitted comparison of these variables between the distinct cohorts. The dataset could have been more reliable had the target location remained constant and only the treatment paradigm changed. Longer follow-up for cohort 1 may have allowed more late failures to emerge; shorter cohort 2 follow-up times could have missed late events. Follow-up was notably less compliant during the COVID pandemic period, which affected cohort 2 specifically. Reliance on electronic medical records to report pain recurrence could have missed subtle pain flare-ups between visits, but the nature of trigeminal pain recurrence is typically severe and progressive, so it is unlikely that significant events would have resolved spontaneously or not been subsequently reported.

## Conclusions

Volumetric SRS prescription to a lower dose/longer segment of the trigeminal nerve and isocentric prescription to a higher dose/tighter volume are both acceptable options for the treatment of trigeminal neuralgia. Additional studies should be pursued to optimize dose volumetric coverage of the trigeminal nerve for radiosurgical treatment of trigeminal neuralgia.
